# The Construction Model of the TCM Clinical Knowledge Coding Database Based on Knowledge Organization

**DOI:** 10.1155/2022/2503779

**Published:** 2022-01-17

**Authors:** Pan Zhang, Shaowu Shen, Wenping Deng, Shusong Mao, Yan Wang

**Affiliations:** ^1^College of Clinical Medicine, Hubei University of Chinese Medicine, Wuhan 430065, China; ^2^College of Information Engineering, Hubei University of Chinese Medicine, Wuhan 430065, China; ^3^Hubei Provincial Hospital of Traditional Chinese Medicine, Wuhan 430065, China

## Abstract

Based on the knowledge organization method, this paper explores the construction method of the traditional Chinese medicine (TCM) clinical knowledge coding model by taking TCM clinical electronic medical record data as the research object. Firstly, extracting technology is used to obtain the required data in the electronic medical record. Then, by constructing the clinical knowledge coding model, the tacit knowledge is made explicit, establishing the clinical knowledge base and exploring the connotation of TCM clinical knowledge. It provides necessary data resources for deepening the expression level of TCM clinical knowledge, constructing accurate TCM clinical diagnosis, intervention, and evaluation models, and promoting the inheritance, innovation, and development of TCM. In this paper, we extracted the data of 318 cases of distention and established the TCM clinical database from the basic information of patients, clinical diagnosis information, clinical diagnosis and treatment information, and clinical evaluation information. Based on the knowledge coding model and the connotation of knowledge attributes, the established TCM clinical knowledge base was to explore the law of TCM clinical precision diagnosis and treatment.

## 1. Introduction

With the development of technology, TCM hospitals at all levels across the country have realized the informatization management of clinical medical records and accumulated a large amount of TCM clinical data. These data are valuable strategic resources for the modernization of TCM hospitals. In June 2016, the General Office of the State Council issued the “Guiding Opinions on Promoting and Regulating the Application and Development of Health and Medical Big Data” and pointed out that health and big medical data are an essential fundamental strategic resource of the country [1]. Managing and making good use of these resources with the help of emerging technologies, serving the inheritance, innovation, and development of traditional Chinese medicine, and realizing the modernization of hospital extensive data governance capabilities have become an urgent problem that needs to be solved.

Electronic medical record (EMR) is an integral part of modern literature, and it records the whole patient diagnosis and treatment process entirely and in detail [2]. It is a medical document with legal effect. They are nonstandardized data that meet the needs of medical business management and cannot be directly studied. Therefore, in conducting clinical research, it is necessary to establish the data required by the research based on the electronic medical record data. With the emergence of new concepts such as evidence-based medicine, real world, and the continuous deepening of research, the field of Chinese medicine has changed from the initial exploration of expert systems [3] to real-world clinical research. For example, Liu proposed a real-world clinical research paradigm of traditional Chinese medicine, a scientific research paradigm that integrates clinical research from the clinic to the clinic [4]. Zhou et al. [5], Gao et al. [6], and other professors studied the prescription medication rules and experienced the inheritance of famous traditional Chinese medicine doctors based on the real world. The types of research knowledge bases increase accordingly, such as Chinese medicine ontology, literature, and clinical research thematic knowledge base. However, most of its databases are based on hospital electronic medical records or expert experience. Most of them stay in the exploration stage of theoretical methods and conceptual levels, unable to present the connotative attributes of knowledge and give full play to the advantages of TCM [7, 8].

Knowledge organization (KO) is based on knowledge as the object through a series of structured processes and methods such as sorting, processing, and presentation, revealing the relationship between the essence of things to order the disorderly scattered knowledge [9]. TCM has unique and complex characteristics, and electronic medical records are primarily unstructured texts described in natural language. Based on the knowledge organization method and natural language processing technology, the clinical knowledge coding model was established to make explicit knowledge in electronic medical records and facilitate the expression and sharing of knowledge [10], to reveal the relationship, and to realize the reconstruction of knowledge and maximize its value. It provides necessary data resources for constructing accurate TCM clinical diagnosis, intervention, and evaluation models.

## 2. Construction Process and Method of the TCM Clinical Knowledge Model

**Figure 1:**
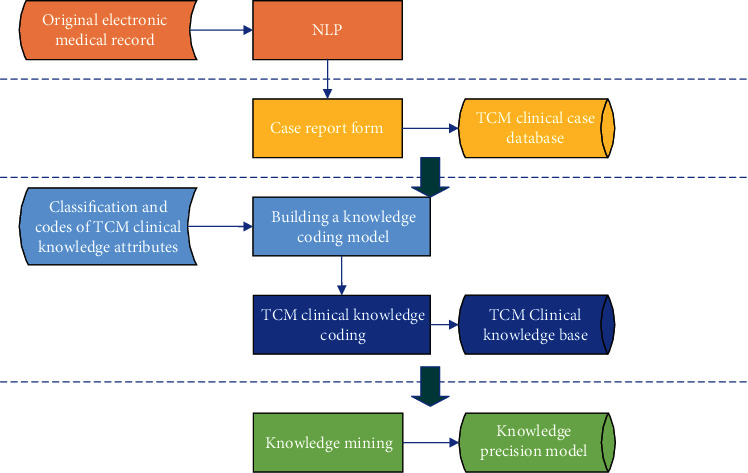
Construction process of the TCM clinical knowledge coding database.

Based on the theory of knowledge organization, they are using NLP technology and using TCM clinical data as the object to study the method of constructing a TCM clinical knowledge model. The construction process of the TCM clinical knowledge coding database is shown in Figure 1. Firstly, natural language extraction technology extracts electronic medical record data. Then, TCM the clinical case knowledge base was established. It can carry out knowledge association, draw a knowledge graph, and establish a precision diagnosis and treatment model. Advanced technologies such as machine reasoning and knowledge discovery are widely used in scientific research, clinical, teaching, and other fields to assist clinicians in solving complex problems and improve work efficiency.

### 2.1. Construction of the TCM Electronic Medical Record Extraction Model

Deep learning neural networks can automatically generate features required for entity recognition tasks, saving a lot of manual screening work. This paper uses the commonly used BiLSTM-CRF and BERT model [11] for named entity recognition (NER) [12]. Data are cleaned and preprocessed (such as data desensitization and screening); then, the named entity corpus is constructed, text feature training is performed on processed data, text features are extracted and labeled, and the information extraction model is constructed using BILSTM-CRF and BERT technology. By setting model parameters and constantly optimizing the model, the specific information is automatically extracted, and the data format is unified to form the structured data that the computer can recognize.

### 2.2. Construction of the TCM Clinical Case Database

According to the traditional Chinese medicine clinical electronic medical record extraction model, the original medical record is transformed into a structured case report form (CRF). According to the taxonomy method and theory and clinical diagnosis and treatment activities, the formed CRF is divided into four parts, namely, basic information, clinical diagnosis information, and clinical treatment information. And the four parts of evaluation information are used to guide and standardize data processing and to establish a TCM clinical case database. The main contents are as follows:

1. CRF-1: it mainly includes basic information of clinical patients, such as medical record number and gender
2. CRF-2: it mainly includes basic information of clinical diagnosis (syndrome differentiation), such as symptoms, diagnosis of traditional Chinese medicine, and diagnosis of western medicine
3. CRF-3: it mainly includes basic information of clinical treatment (treatment), such as Chinese medicine prescriptions and western medicine prescriptions
4. CRF-4: it mainly includes basic information of clinical evaluation, such as the dynamic changes and evaluation of symptoms, tongue diagnosis, and pulse diagnosis

### 2.3. Construction of the Coding Model of TCM Clinical Knowledge

**Figure 2:**
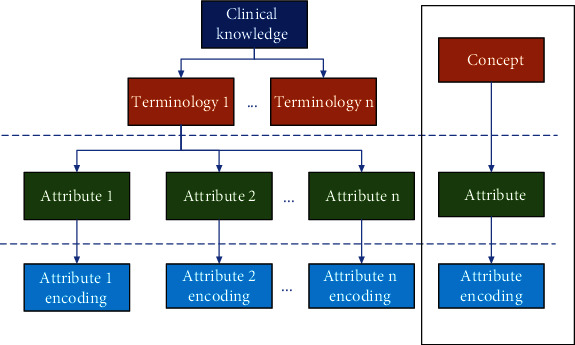
Knowledge coding structure.

Knowledge coding, that is, knowledge attribute coding, is an effective way of knowledge management, expressing knowledge in standard coding, so that knowledge can be easily shared and exchanged. Extract the clinical feature information of the clinical diagnosis and treatment knowledge in the CRF, analyze the attributes, and encode each clinical diagnosis and treatment attribute according to the coding standard of TCM clinical knowledge to describe the clinic more entirely and accurately.  Figure 2 shows the knowledge coding structure.

#### 2.3.1. Terminology Specification

Due to the characteristics of individualized TCM clinical practice, the knowledge system of TCM is complex, and the language forms used to express information are complex and diverse. Different clinicians often use different expression methods. Many terms have multiple meanings and words, resulting in symptomatic terms—the phenomenon of nonuniformity and irregular use. For example, the symptom “stomach ache” has different descriptions. Therefore, the terminology extracted from the description of TCM clinical knowledge shall be standardized concerning the *Terms of Traditional Chinese Medicine* and *Terms of Clinical Diagnosis and Treatment of Traditional Chinese Medicine* published by the TCM Term Verification Committee to realize the standardized expression of TCM knowledge and improve the level of standardization of TCM clinical knowledge description, collection, processing, analysis, retrieval, and the consistency and accuracy of association.

#### 2.3.2. Classification of Knowledge Attributes

After standardizing and unifying the terms, according to the TCM clinical knowledge attribute coding standard, the connotation attribute of each term is extended from the conceptual level to the attribute level, the ontology structure of TCM knowledge and its inherent complex logical relationship is clarified, and the process or behavior of TCM knowledge is explored. For example, the perilla in the prescription of TCM can be developed from the connotative attributes, which can be divided into four qi, five flavors, meridian, ups and downs, functions, indications, compatibility taboos, etc.

#### 2.3.3. Knowledge Attribute Coding

Knowledge attribute coding is a process of structuring and digitizing explicit knowledge and tacit knowledge, especially the explicit and coding of tacit knowledge, so that tacit knowledge becomes codeable knowledge, easy to transfer and share, and easy for computers to receive and share. The way of processing embodying description logic is conducive to collecting, mining, processing, sharing, and utilizing Chinese medicine knowledge by computers. The process of knowledge coding is not a simple process of knowledge expression but a process of knowledge reorganization and recreation.

**Figure 3:**
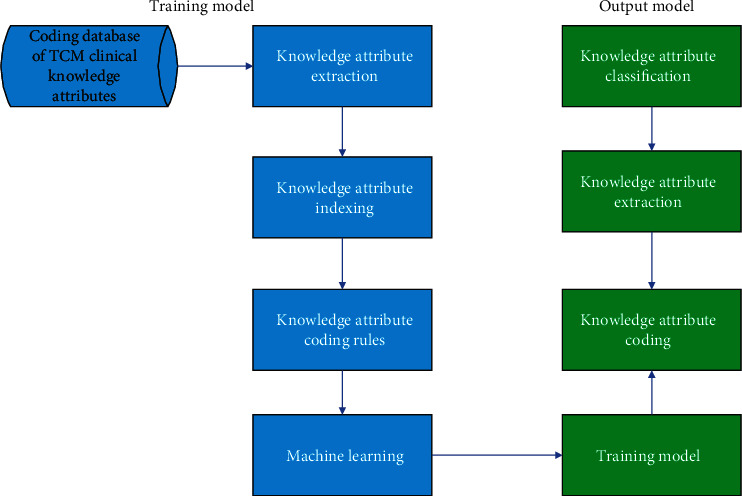
Knowledge coding model.

According to the TCM clinical knowledge attribute coding standard, the TCM clinical knowledge attribute coding database is established, the characteristics of the knowledge attribute are extracted for indexing, and the knowledge coding training model is established using machine learning according to the coding rules to encode different knowledge attributes.  Figure 3 shows the knowledge coding model.

### 2.4. Construction of the Knowledge Coding Database

The knowledge base is a database organized by extracting knowledge attributes from knowledge in a targeted manner based on ordinary databases to form a knowledge system for sorting and analyzing. It is a collection of knowledge after classification, ordering, and reorganization. Through the classification and coding of knowledge attributes, TCM clinical knowledge coding database is established, TCM clinical data attributes are reconstructed, tacit knowledge in clinical cases is realized, and the connotation of TCM clinical case knowledge is enriched.

### 2.5. Construction of the Coding Model of TCM Clinical Knowledge

With the rapid development of big health data, the rapid growth of multisource heterogeneous TCM information and data poses severe challenges to knowledge organization and knowledge expression in TCM. With the help of data mining, machine learning, and other technologies, using the knowledge organization method to research the TCM clinical knowledge base, it can conduct correlation analysis of TCM clinical knowledge and build accurate TCM clinical diagnosis, and treatment models optimize clinical models. Through drawing TCM clinical knowledge maps and revealing TCM clinical knowledge, the facts and laws of the Chinese medicine guide clinical practice and form a closed-loop feedback control system of “from the clinic to the clinic” providing necessary technical support for the development of the clinical artificial intelligence (AI) system of Chinese medicine.

## 3. Case Analysis

### 3.1. Data Collection

This article extracts the inpatient electronic medical record data of patients with wind dropsy disease in a Chinese hospital from April 2017 to May 2019. The selection criteria are that the primary diagnosis is wind dropsy disease. The cases with complete information and treatment based on TCM have selected 318 cases. In order to ensure the privacy of patients and the security of information, the information has been desensitized.

### 3.2. Data Extraction

In order to ensure a comprehensive and accurate record of the whole process of disease occurrence, the required data were extracted from the basic patient information, clinical diagnosis, clinical treatment, and clinical evaluation, and the original medical record data were transformed into structured CRF. When extracting part of the data, to ensure the authenticity of the data, the information of the missing items was retained, and finally, the TCM clinical bloat disease database was established.

Taking the extraction of clinical diagnosis information as an example, according to the admission records, disease course records, and discharge records in the electronic medical record, it is extracted into the structured CRF diagnosis information. The extraction model first expresses the sentence as a vector through pretrained word embedding and then learns the context-related vector through a representation such as a convolutional neural network and finally generates a sequence label through a decoder such as a conditional random field to perform named entity recognition. The specific extraction examples are as follows:

1. Input: {original electronic medical record data}
2. Word vector conversion: *x* = (*x*1, *x*2, ⋯, *xn*), x*i* ∈ *R*^d^
3. Pretraining or random initialization: $\left(\overset{⟶}{h}1,\overset{⟶}{h}2,\cdots ,\overset{⟶}{h}t\right)$$\left(\overset{⟵}{h}1,\overset{⟵}{h}2,\cdots ,\overset{⟵}{h}n\right)$
4. LSTM backward overlaps the previous term: $\text{ht}=\left[\overset{⟶}{h}t;\overset{⟵}{h}t\right]\in R^{\text{m}}$
5. LSTM output: (*h*1, *h*2, ⋯, *hn*) ∈ *R*^*n*∗*m*^
6. Conditional random field layer: *p* = (*p*1, *p*2, ⋯, *pn*) ∈ *R*^*n*∗*k*^, *pi* ∈ *R*^k^
7. Output: {Main complaint:}, {History of present illness:}, {Inscribing symptoms:}, {Past history:}, {History of allergy:},…,{Name of disease in Chinese medicine:}, {Syndrome of Chinese medicine}, {Name of disease in Western medicine:}…

### 3.3. TCM Clinical Knowledge Coding

After the TCM clinical bloat disease database is established, the clinical knowledge is expanded from the conceptual level to the attribute level for coding according to the knowledge coding model. For example, the symptoms are expanded to 25 attributes, the tongue diagnosis is expanded to 8 attributes, and the pulse diagnosis is expanded to 3 attributes, the prescription information of traditional Chinese medicine is expanded to 16 attributes, and finally, the clinical knowledge base is established. For example, the symptom specifications refer to the symptom standard corpus established by *Diagnosis of Traditional Chinese Medicine* and *Terms of Traditional Chinese Medicine*, and the symptom attribute coding refers to the corpus established by *Classification and Code of Basic Clinical Symptom Information of Traditional Chinese Medicine* (TCIATCM 020-2019).

1. Enter the symptom description: severe fatigue, body pain, spontaneous sweating, coughing white and sticky sputum, hiccups after eating, nausea, anorexia, low stool, and yellow urine
2. Symptom criteria: {severe fatigue}, {body pain}, {spontaneous sweating}, {coughing white sticky sputum}, {hiccup}, {nausea}, {lack of food}, {little stools}, and {yellow urine}
3. Coding of symptom knowledge attribute: {ZZ066104.P3}, {ZZ004103.A9901}, {ZZ003102.C138}, {ZZ22430104.C035D300H10}, {ZZ22530105}, {ZZ057106}, {ZZ08110602}, {ZZ09510701.J2}, and {ZZ0962030402.D200}

### 3.4. Accurate Model of TCM Clinical Knowledge

After completing the knowledge coding, the knowledge base of TCM clinical inflation disease was established. The analysis method in data mining was used to establish a deep association rule mining model for association analysis, and the complex relationship and internal rules of “syndrome-symptom-syndrome” were found from different perspectives, providing clinical guidance for subsequent disease treatment programs.

## 4. Result

In this paper, 319 cases of electronic medical records of distention in TCM hospitals and 318 cases of compelling data are selected to establish a clinical distention database, extend and encode the connotation of knowledge attribute from clinical diagnosis information and clinical treatment information, and establish the knowledge coding database of clinical distention of TCM.

### 4.1. Clinical Diagnostic Information Knowledge Coding Library

According to the TCM clinical knowledge coding model, the clinical diagnosis information automatically extracted was expanded to build the clinical diagnosis knowledge library by the connotation attributes of symptoms, tongue image, pulse image, and other diagnostic information, as shown in Tables 1–3.

### 4.2. Clinical Treatment Information Knowledge Coding Library

According to the clinical knowledge coding model of TCM, the clinical treatment information automatically extracted was expanded to build the clinical treatment information knowledge database. Some information is shown in Table 4.

## 5. Discussion

The knowledge attribute connotation of the database is extended and coded, and the knowledge base of clinical distention is established. At present, the computer-aided named entity recognition and knowledge coding are limited mainly by the quantity and quality of the trained corpus and the complexity of the knowledge relationship of TCM. They are usually carried out synchronously by manual verification. In the later stage, the quantity and quality of corpus will be expanded and improved, the accuracy of the training model will be improved, and the problems of labor time and efficiency will be solved to a great extent. In this paper, through the study of the clinical knowledge coding database, knowledge attribute coding is applied to the original clinical data for knowledge attribute reconstruction and structural and data processing to make the clinical tacit knowledge explicit. The connotation of clinical knowledge is revealed through knowledge mining, and the level of clinical knowledge expression is deepened. It is the basis for realizing the precision of clinical diagnosis, intervention, and evaluation and also provides data support for clinical extensive data knowledge engineering research.

## 6. Conclusion

The emergence of new technologies such as big data, knowledge engineering, Internet, Internet of things, and artificial intelligence has driven the change of TCM clinical research mode and method, promoted the reconstruction of TCM clinical research technology system, overturned the innovation and development of TCM clinical research, and had a significant and far-reaching impact on the modernization of TCM. Therefore, in the future knowledge base construction and research, on the one hand, we should pay attention to the research of corpus and other fundamental work; on the other hand, we should make full use of the current advanced technology and methods to optimize the model to improve the intelligence level of knowledge base, to promote the inheritance and utilization of TCM.

## Figures and Tables

**Table 1:** Symptom information knowledge coding.

Symptom	Code	Key symptoms	Access	Body part	Nature	Color	Output	…
Body pain	ZZ004103.A9901	004	10300000	A99010000				
Spontaneous perspiration	ZZ003102.C138	003	10200000		C138			
Hiccup	ZZ22530105	225	30105000					
Nausea	ZZ057106	057	10600000					
Stool quantity is little	ZZ09510701.J2	095	10701000				J2	
Dark urine	ZZ0962030402.D200	096	20304020			D200		

**Table 2:** Tongue diagnostic knowledge coding.

Tongue diagnosis	Code	Tongue color	Ligulate	Coating color	Fur character	Tongue nature	…
Tongue thin fat big, the moss is yellow and thick and greasy	SZA200060000, TB1000900.Z22	A2	06	B1	09	22	
Tongue pale red, moss thin yellow	SZC200000000, TB1000800	C2		B1	08		
Tongue pale red, moss thin white	SZC200000000, TA1000800	C2		A1	08		
Tongue red, moss white and greasy	SZC100000000, TA1000900.Z22	C1		A1	09	22	
Tongue pale red, moss thin yellow	SZC200000000,TB1000800	C2		B1	08		

**Table 3:** Pulse diagnostic knowledge coding.

Pulse diagnosis	Code	Pulse1	Pulse2	Pulse3	Degree of pulse	Division of pulse
Thin and string pulse	MZ53640000	53	64			
String and slippery pulse	MZ62640000	62	64			
String pulse	MZ64000000	64				

**Table 4:** Chinese herbal medicine knowledge coding.

Chinese herbal medicine	Code-GB	Four qi	Five flavours1	Five flavours2	Effect	Toxicity	…
Bupleurum	06164310101003008	YX0103	YX0204	YX0202	YX0601001	YX0304	
White peony	06153710100202008	YX0103	YX0202	YX0201	YX0619003	YX0304	
Poria	06400210100403009	YX0110	YX0203	YX0206	YX0606001	YX0304	
Amomum	06193540200300001	YX0106	YX0204		YX0605	YX0304	
Salvia	06172210300103006	YX0103	YX0202		YX0612002	YX0304	

## Data Availability

The data used in this paper are all from electronic medical records of hospitals.
